# Human immune deficiency virus among cervical cancer patients at Tikur Anbessa Specialized Hospital, Ethiopia: a cross sectional study

**DOI:** 10.1186/s12905-021-01438-7

**Published:** 2021-08-09

**Authors:** Mulugeta Wassie, Beletech Fentie, Tseganesh Asefa

**Affiliations:** 1 Department of Medical Nursing, School of Nursing, College of Medicine and Health Sciences, University of Gondar, Gondar, Ethiopia; 2 Department of Pediatrics and Child Health Nursing, School of Nursing, College of Medicine and Health Sciences, University of Gondar, Gondar, Ethiopia

**Keywords:** HIV, Cervical cancer, TASH, Ethiopia

## Abstract

**Background:**

The discrepancy in cervical cancer incidence between women with HIV and women without HIV is highest in low and middle-income countries. In Africa, cervical cancer is the most common cause of cancer death. As a result, HIV-infected women are 6 times more likely to develop cervical cancer than uninfected women. In addition, HIV is associated with several triggering factors for cervical cancer, including multiple sexual partners, early sexual debut, economic status and substance use.

**Objective:**

To assess the prevalence and associated factors of HIV among cervical cancer patients at Tikur Anbessa Specialized Hospital, Addis Ababa, Ethiopia.

**Methods:**

A cross sectional study was conducted among 1057 cervical cancer patients registered from January 1, 2014 to December 31, 2018 at Oncology Center of Tikur Anbessa Specialized Hospital. A structured English version checklist was used to collect the data from patient charts. The pre coded data were entered in to EPI-data version 3.1 then exported to STATA version 14.0 for analysis. Both bivariable and multivariable regression analysis were carried out. Variables with *p* value < 0.05 in multivariable logistic regression were consider as significant predictors of the outcome variable.

**Result:**

The prevalence of HIV among cervical cancer patients was 18.35%. HIV among cervical cancer patients was significantly associated with age group 30–39 [AOR = 2.83; 95%CI (1.27, 6.22)] and 40–49 [AOR = 2.39; 95%CI (1.07, 5.32)], employed [AOR = 2.23; 95%CI (1.46, 3.41)] and substance users [AOR = 3.92; 95%CI (2.04, 6.28)].

**Conclusion:**

This study revealed that about 18% of cervical cancer patients were HIV seropositive. HIV seropositivity was significantly increased with 30–49 age group, employed and substance users. Authors recommended that it is better to screen all HIV seropositive patients for cervical cancer and give greater attention for women with cervical cancer in the age groups of 30–49 years, employed and substance users.

## Introduction

The prevalence of Human Immunodeficiency Virus (HIV) in women has suddenly increased since the early 1980s, when the diseases first entered public consciousness. Today an estimated 18 million women are living with HIV, in place of more than half of HIV-seropositive adults worldwide [1].

People living with HIV have been shown higher rates of HPV infection and more likely to be infected with high risk HPV and multiple HPV types than HIV negative individuals [2]. Women living with HIV have a greater incidence of Human Papilloma Virus (HPV) infection than do the general population. Numerous studies revealed that immune suppression with low CD4 counts predisposes women to high risk of HPV infection. HPV infection is the principal cause of oncogenic transformation and development of cervical intraepithelial neoplasia (CIN), which are precursor lesions for cervical cancer [2, 3].

The discrepancy in cervical cancer incidence between women with HIV and without HIV is greatest in low and middle-income countries. In Africa, cervical cancer is the leading cause of cancer death, in which HIV-infected women are about 6 times more likely than uninfected women to develop cervical cancer death. In addition, HIV is associated with several enabling factors for cervical cancer, including multiple sexual partners, early sexual debut, economic status and substance use [4, 5].

Hence, HPV infection associated to HIV status is not the exclusive cause of cervical cancer. In addition to HIV associated HPV infection, other factors such as parity, smoking, diet, physical inactivity, sexual behavior, use of oral contraceptives, and aging are also contribute to the development of cervical cancer [6]. Therefore, this study aimed to assess the prevalence and associated factors of HIV among cervical cancer patients at TASH, Ethiopia.

## Method and materials

### Study design, period and setting

A cross sectional study was conducted at oncology center of TASH from March to April 2019. The hospital was established in 1972 and has more than 800 beds providing diagnostic and treatment service for about 400,000 patients per year. The oncology center at TASH is the largest referral site in the country, providing service for over 60,000 patients annually. It is the sole oncology referral and radiotherapy center in Ethiopia [7].

### Study population

Medical records of women diagnosed with cervical cancer from January 1, 2014 to December 31, 2018 at oncology center of TASH were a study populations. Incomplete medical chart record and charts not found during a data collection were excluded.

### Sample size determination and sampling procedure

All medical records of patients diagnosed with cervical cancer from January 1, 2014 to December 31, 2018 was the total sample size. A medical record of 1057 cases fulfilled the inclusion criteria. HIV status of the patient was the dependent variable, the patient HIV status was retrieved by trained medical staff from medical record chart and grouped HIV status as HIV-positive and HIV-negative. Sociodemographic characteristics such as age, marital status, resident, region, religion, occupation, substance use, number of children, stage, histopathology, anemia and treatment initiated were independent variables.

### Operational definition

Substance use: Patients who used one, two or all of the three substances (cigarate, chat and alcohol) [8].

### Data collection tools and quality assurance

A structured English version checklist was used to collect the data from patient charts. The checklist contains sociodemographic factors and HIV status of cervical cancer patients. Prior to data collection, training was provided for three data collectors having bachelors of degree in nursing and two supervisors having master’s degree in clinical oncology nursing about the checklist and how to retrieve data from the medical record.

Pre-taste was conducted in 5% of the total sample size and the necessary modification was made on checklist accordingly. Furthermore, the data collection process was strictly supervised for completeness of the data before chart returned to the shelf.

### Data processing and analysis

The pre coded data were entered in to EPI-data version 3.1 then exported to STATA version 14.0 for analysis. The descriptive statistics were used to describe the study population related to different characteristics. Binary logistic regression was employed for each variables with the outcome variable and those variables with *p* < 0.2 were entered to multivariable analysis. Variables with *p* value < 0.05 in multivariable logistic regression were considered as a significant predictor of HIV among cervical cancer patients.

## Results

### Socio-demographic characteristics of study participants

In this study, 1057 cervical cancer patients were surveyed. Two hundred sixty two (24.79%) of participants were under the age category 40–49 years. Six hundred sixty five (62.91%) of participants were married. Six hundred fourteen (58.09%) of respondents came from urban area.

By region, 281 (26.58%) and 280 (26.49) of participants were from Amhara and Addis Ababa region, respectively. Six hundred twenty nine (59.51%) of respondents were orthodox Christianity followers in their religion. More than 905 (85.62%) and 875 (82.78%) of participants were unemployed and substance non user, respectively. Four hundred forty (41.63%) of participants have more than three children. More than half (56.76%) of participants were diagnosed at advanced (stage III & IV). Closely half (51.18%) and (49.20%) of patients were anemic and received radiation therapy, respectively (Table 1).

**Table 1:** Socio demographic and clinical characteristics of study participants at TASH, Ethiopia (n = 1057)

Characteristics	Code	Frequency	Percent %
Age	< 30	92	8.70
	30–39	219	20.72
	40–49	262	24.79
	50–59	245	23.28
	> = 60	239	22.61
Marital status	Single	392	37.09
	Married	665	62.91
Residence	Urban	614	58.09
	Rural	443	41.91
Region	Amhara	281	26.58
	Oromia	341	32.26
	Tigray	30	2.84
	SNNP	95	8.99
	Addis Ababa	280	26.49
	Others	30	2.84
Religion	Orthodox	629	59.51
	Muslim	201	19.02
	Protestant	212	20.06
	Other	15	1.42
Occupation	Unemployed	905	85.62
	Employed	152	14.38
Substance use	User	182	17.22
	None user	875	82.78
Number of children	No child	40	3.78
	One child	69	6.53
	Two child	147	13.91
	Three child	361	34.15
	> 3 child	440	41.63
Stage	Stage I & II	457	43.24
	Stage III & IV	600	56.76
Histopathology	Squamous cell carcinoma	959	90.73
	Adenocarcinoma	98	9.27
Anemia	Yes	541	51.18
	No	516	48.82
Treatment initiated	Surgery and chemo	17	1.61
	Chemo	19	1.80
	Radiotherapy	520	49.20
	Chemo and RT	375	35.48
	Surgery and RT	111	10.50
	Chemo + surgery + RT	15	1.42

### Prevalence of HIV among cervical cancer patients

**Fig. 1:**
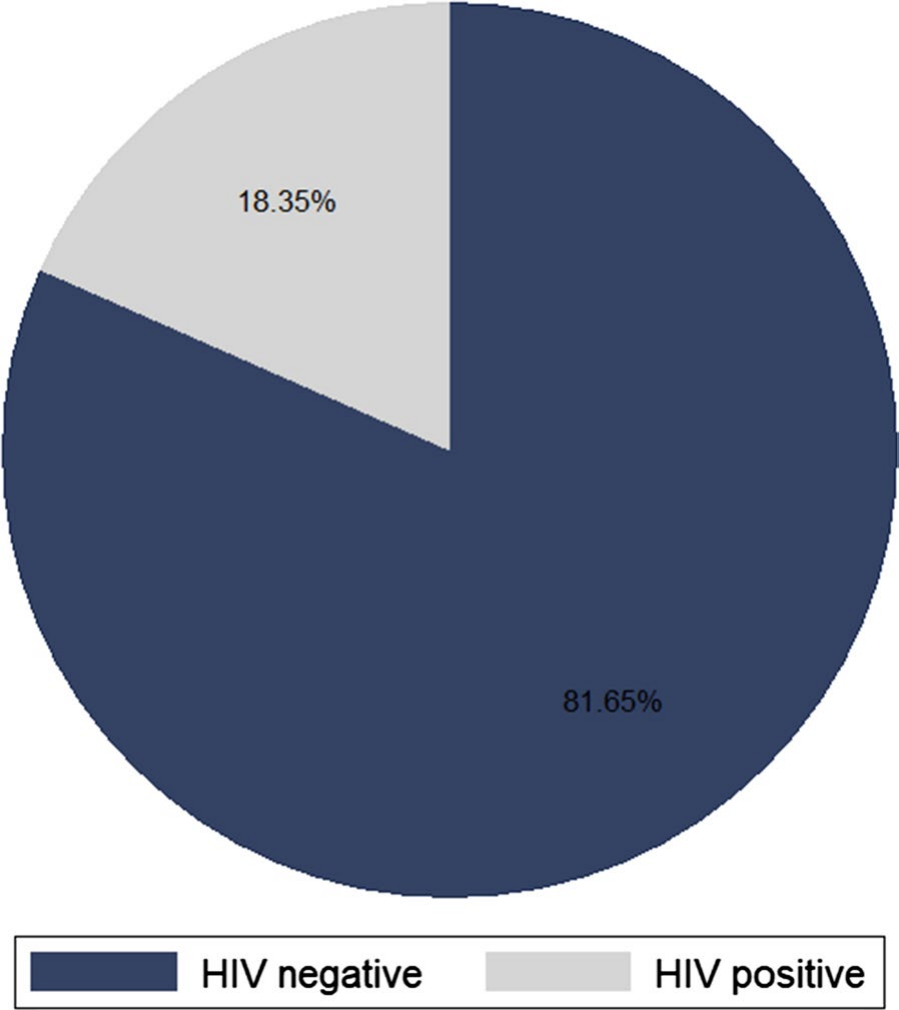
Prevalence of HIV among cervical cancer patients at TASH, Ethiopia (n = 1057)

The overall prevalence of HIV among cervical cancer patients was 18.35% [95% CI (0.16, 0.21)] (Fig. 1). HIV prevalence was higher (27.84%) among 40–49 age groups of cervical cancer patients. Nearly three fifth (57.73%) of married cervical cancer patients were HIV positive. Closely two third (65.46%) of cervical cancer patients live in urban area were HIV positive. More than two third (35.57%) of cervical cancer patients from Addis Ababa were HIV positive. Whereas three fourth (74.74%) of unemployed cervical cancer patients were HIV positive. About two third (63.40%) and (61.86%) of cervical cancer patients who did not use substances and diagnosed at advanced stage (III & IV) were HIV positive, respectively. Nearly two fifth (37.63%) and (44.85%) of cervical cancer patients, who have three children and received a chemo/RT were HIV positive. Three fourth (76.8%) of anemic cervical cancer patients were HIV positive (Table 2).

**Table 2:** Prevalence of HIV among cervical cancer patients at TASH, Ethiopia (n = 1057)

Characteristics	HIV	
	Positive N (%)	Negative N (%)
*Age*		
< 30	9 (4.64)	83 (9.62)
30–39	52 (26.80)	167 (19.35)
40–49	54 (27.84)	208 (24.10)
50–59	46 (23.71)	199 (23.06)
> = 60	33 (17.01)	206 (23.87)
*Marital status*		
Single	82 (42.27)	310 (35.92)
Married	112 (57.73)	553 (64.08)
*Residence*		
Urban	127 (65.46)	487 (56.43)
Rural	67 (34.54)	376 (43.57)
*Region*		
Amhara	49 (25.26)	232 (26.88)
Oromia	44 (22.68)	297 (34.41)
Tigray	7 (3.61)	23 (2.67)
SNNP	22 (11.34)	73 (8.46)
Addis Ababa	69 (35.57)	211 (24.45)
Others	3 (1.55)	27 (3.13)
*Religion*		
Orthodox	113 (58.25)	516 (59.79)
Muslim	31 (15.98)	170 (19.70)
Protestant	49 (25.26)	163 (18.89)
Other	1 (0.52)	14 (1.62)
*Occupation*		
Unemployed	145 (74.74)	760 (88.06)
Employed	49 (25.26)	103 (11.94)
*Substance use*		
User	71 (36.60)	111 (12.86)
None user	123 (63.40)	752 (87.14)
*Number of children*		
No child	10 (5.15)	30 (3.48)
One child	25 (12.89)	44 (5.10)
Two child	35 (18.04)	112 (12.98)
Three child	73 (37.63)	288 (33.37)
> 3 child	51 (26.29)	389 (45.08)
*Stage*		
Stage I&II	74 (38.14)	383 (44.38)
Stage III &IV	120 (61.86)	480 (55.62)
*Histopathology*		
Squamous cell carcinoma	181 (93.30)	778 (90.15)
Adenocarcinoma	13 (6.70)	85 (9.85)
*Anemia*		
Yes	149 (76.8)	392 (45.42)
No	45 (23.20)	471 (54.58)
*Treatment initiated*		
Surgery and chemo	5 (2.58)	12 (1.39)
Chemo	1 (0.52)	18 (2.09)
Radiotherapy	79 (40.72)	441 (51.10)
Chemo and RT	87 (44.85)	288 (33.37)
Surgery and RT	21 (10.82)	90 (10.43)
Chemo + surgery + RT	1 (0.52)	14 (1.62)

### Predictors of HIV among cervical cancer patients

Age, marital status, residence, region, occupation, substance use and number of children were fitted the outcome variable in the bivariable binary logistic regression at *p* < 0.2. All variables with *p* < 0.2 in the bi-variable analysis were included in the multivariable analysis. In multivariable analysis, three variables were found to be statistically significant at *p* < 0.05 with 95% confidence interval. Accordingly, age, occupation and substance use demonstrated to have statically significant association with HIV positive among cervical cancer patients.

Cervical cancer patients aged 30–39 were 3 times (AOR = 2.83; 95% CI = 1.27, 6.32) more likely to be HIV seropositive than their counter parts. Correspondingly, cervical cancer patients aged 40–49 were two times (AOR = 2.38; 95% CI = 1.07, 5.32) more likely to be HIV seropositive than their counter parts. Employed cervical cancer patients were two times (AOR = 2.24; 95% CI = 1.46, 3.44) more likely to be HIV seropositive than unemployed. Substance user cervical cancer patients were four times (AOR = 3.93; 95% CI = 2.64, 5.83) more likely HIV seropositive than non-users (Table 3).

**Table 3:** Result of bivariable and multivariable binary logistic regression analysis of cervical cancer patients at TASH, Ethiopia (n = 1057)

Characteristics	HIV positive N (%)	HIV negative N (%)	COR (95% CI)	AOR (95% CI)	P value
**Age**					
< 30	9 (4.64)	83 (9.62)	1	1	
30–39	52 (26.80)	167 (19.35)	2.87 [1.34, 6.11]	2.83 [1.27, 6.32]	0.011*
40–49	54 (27.84)	208 (24.10)	2.39 [1.13, 5.07]	2.38 [1.07, 5.32]	0.034*
50–59	46 (23.71)	199 (23.06)	2.13 [0.99, 4.55]	2.13 [0.94, 4.84]	0.070
> = 60	33 (17.01)	206 (23.87)	1.48 [0.68, 3.22]	1.37 [0.59, 3.17]	0.457
**Marital status**					
Single	82 (42.27)	310 (35.92)	1.31 [0.95, 1.79]	1.35 [0.95, 1.92]	0.087
Married	112 (57.73)	553 (64.08)	1	1	
**Residence**					
Urban	127 (65.46)	487 (56.43)	1	1	
Rural	67 (34.54)	376 (43.57)	0.68 [0.49,0.94]	1.02 [0.67, 1.55]	0.929
**Region**					
Amhara	49 (25.26)	232 (26.88)	1	1	
Oromia	44 (22.68)	297 (34.41)	0.70 [0.45, 1.09]	0.74 [0.45, 1.18]	0.209
Tigray	7 (3.61)	23 (2.67)	1.44 [0.58, 3.55]	1.70 [0.64, 4.49]	0.284
SNNP	22 (11.34)	73 (8.46)	1.43 [0,81, 2.52]	1.76 [0.95, 3.26]	0.071
Addis Ababa	69 (35.57)	211 (24.45)	1.55 [1.03, 2.34]	1.47 [0.90, 2.41]	0.121
Others	3 (1.55)	27 (3.13)	0.53 [0.15, 1.80]	0.49 [0.14, 1.79]	0.286
**Occupation**					
Unemployed	145 (74.74)	760 (88.06)	1	1	
Employed	49 (25.26)	103 (11.94)	2.49 [1.69, 3.66]	2.24 [1.46, 3.44]	0.000*
**Substance use**					
User	71 (36.60)	111 (12.86)	3.91 [2.75, 5.57]	3.93 [2.64, 5.83]	0.000*
None user	123 (63.40)	752 (87.14)	1		
**Number of children**					
No child	10 (5.15)	30 (3.48)	1	1	
One child	25 (12.89)	44 (5.10)	1.70 [0.72, 4.06]	2.22 [0.83, 5.91]	0.111
Two child	35 (18.04)	112 (12.98)	0.94 [0.42, 2.11]	1.38 [0.56, 3.44]	0.485
Three child	73 (37.63)	288 (33.37)	0.76 [0.35, 1.63]	1.01 [0.42, 2.43]	0.973
> 3 child	51 (26.29)	389 (45.08)	0.39 [0.18, 0.85]	0.81 [0.33, 1.97]	0.644

CI: confidence interval, * significant at p-value less than 0.05

## Discussion

The current study aimed to assess the prevalence and associated factors of HIV among cervical cancer patients. In this study the prevalence of HIV among cervical cancer patient was about 18%. This prevalence is in line with the study in KwaZulu-Natal, South Africa with the prevalence of 21% [9]. On the other hand, the result of this study is higher than the studies conducted in Ethiopia 13.3% [10], South Africa 7.2% [11], Nigeria 6%[12], Kenya 1.5% [13] and Guinea 2.1% [14]. This might be due to variations in the sample size, study period, study area and population.

In the current study, the women within the age group of 30–39 and 40–49 years were about three times and two times more HIV seropositive than those age with age group below 30 years, respectively. This finding is similar with the study done in Ivory Coast [15], Tanzania [16] and Kenya [17]. This might be women with age 30–49 years a time which become sexually active and may have a repeated unprotected sexual exposure that my contribute to increase the rate of HIV prevalence [18]

Substance users presented with HIV seropositive status about four times more than non-user cervical cancer patients. This finding comparable with studies in Brazil [19], USA [20] and South Africa [21]. This could be due to the fact that substance use has been a potential to divert a person attitude towards sexual risk behavior such as unprotected sex and multiple sexual partners [22]. On the other hand, it reduce the immune system ability to fight against infections like HPV in addition it introduce chemicals which might be carcinogens to body [23]. Correspondingly, other studies revealed HIV infection itself as a risk factor for cervical cancer. All this conditions might predispose individuals to HPV infection and facilitate cervical cancer development.

Employed cervical cancer patients in the current study presented with HIV seropositive status about two times higher than those unemployed. This finding is supported by the study in Brazil [24]. Women who works in hazardous environment without protective equipment could contribute to the increment of HIV prevalence for the employed patients [25]. In addition other study stated that HIV infection is associated with some occupations like health care workers like surgeons, anesthetist, and physicians engaged in invasive procedures [26]. This could indicate that employed in unsafe work environment might be associated with HIV infection.

## Conclusion

This study revealed that about 18% of cervical cancer patients were HIV seropositive. HIV sero positivity significantly increased with 30–49 age group, employed and substance users. Authors recommend that it is better to screen all HIV seropositive for cervical cancer and give greater attention for women with cervical cancer in the age groups of 30–49 years employed and substance users.

## Data Availability

The datasets used in this study are available upon of correspondent author.

## Abbreviations

AOR: Adjusted odd ratio
HIV: Human immunodeficiency virus
HPV: Human papilloma virus
OR: Odd ratio
TASH: Tikur Anbessa Specialized Hospital
